# Association between postoperative ibuprofen exposure and acute kidney injury after pediatric cardiac surgery

**DOI:** 10.1080/0886022X.2024.2318417

**Published:** 2024-02-19

**Authors:** Sheng Shi, Chao Xiong, Dongyun Bie, Zhongrong Fang, Jianhui Wang

**Affiliations:** Department of Anesthesiology, National Center of Cardiovascular Diseases, Fuwai Hospital, Chinese Academy of Medical Sciences and Peking Union Medical College, Beijing, China

**Keywords:** Acute kidney injury, cardiac surgery, ibuprofen, pediatrics

## Abstract

**Background:**

Acute kidney injury (AKI) is a common complication after pediatric cardiac surgery and is associated with worse outcomes. Ibuprofen is widely used in the perioperative period and can affect kidney function in children. However, the association between ibuprofen exposure and AKI after pediatric cardiac surgery has not been determined yet.

**Methods:**

In this retrospective cohort study, children undergoing cardiac surgery with cardiopulmonary bypass were studied. Exposure was defined as given ibuprofen in the first 7 days after surgery. Postoperative AKI was diagnosed using the KDIGO criteria. A multivariable Cox regression model was used to assess the association between ibuprofen exposure and postoperative AKI by taking ibuprofen as a time-varying covariate.

**Results:**

Among 1,112 included children, 198 of them (17.8%) experienced AKI. In total, 396 children (35.6%) were exposed to ibuprofen. AKI occurred less frequently among children who were administered ibuprofen than among those who were not (46 of 396 [11.6%] vs. 152 of 716 [21.2%], *p* < 0.001). Using the Cox regression model accounting for time-varying exposures, ibuprofen treatment was not associated with AKI (adjusted HR, 0.99; 95% CI 0.70–1.39, *p* = 0.932). This insignificant association was consistent across the sensitivity and subgroup analyses.

**Conclusions:**

Postoperative ibuprofen exposure in pediatric patients undergoing cardiac surgery was not associated with an increased risk of AKI.

## Introduction

Acute kidney injury (AKI) is a common complication after pediatric cardiac surgery, occurring in up to 52% of children in the early postoperative period [1]. Postoperative AKI is associated with a higher risk of in-hospital mortality, need for kidney replacement therapy, cardiac arrhythmias, longer ventilation time, and hospital length of stay [2]. AKI after pediatric cardiac surgery is mediated by various pathways that affect oxygen demand, inflammation, and microcirculation in the kidneys [3]. Exposure to nephrotoxic medications contributes to a large portion of AKI cases [4]. Nephrotoxins are commonly used in postcardiac surgical intensive care units after pediatric congenital heart surgery. As nephrotoxin exposure represents one of the few modifiable risk factors in this surgical population, more judicious use of these medications might lower the risk of iatrogenic AKI.

Ibuprofen is one of the most commonly used nonsteroidal anti-inflammatory drugs (NSAIDs) for the treatment of pain and fever in children undergoing cardiac surgery. Even correctly dosed NSAIDs have been reported to be associated with an increased risk of AKI in the pediatric population, probably by reducing renal blood flow through the inhibition of prostaglandin-mediated vasodilation [5]. Ibuprofen use has been associated with an increased risk of AKI in children [6,7], but few studies have investigated whether this association remains in children undergoing cardiac surgery. Therefore, this retrospective cohort study was designed to determine the association between ibuprofen exposure and AKI after pediatric cardiac surgery.

## Methods

### Study population and data extraction

We conducted a retrospective observational cohort study of children undergoing cardiac surgery between March 1, 2022, and September 30, 2022, at Fuwai Hospital, Beijing, China. The study was approved by the institutional review board of the Fuwai Hospital. The inclusion criteria were age 1 month to 14 years and use of cardiopulmonary bypass (CPB) during surgery. The exclusion criteria were as follows: (1) failure to determine AKI status due to the absence of perioperative serum creatinine values; (2) ibuprofen exposure within 7 days before surgery; and (3) missing admission data.

Preoperative data including age at operation, sex, weight, cyanosis, prior cardiac surgery, serum creatinine, serum albumin, hemoglobin, and left ventricular ejection fraction (LVEF) were extracted. For laboratory data, the most recently measured levels within 7 days prior to surgery were identified as baseline values. Intraoperative data, including the Risk Adjustment for Congenital Heart Surgery 1 (RACHS-1) category, emergency surgery, CPB duration, and cross-clamp time, were collected.

### Exposure and outcome definitions

Exposure was defined as time-varying dichotomous exposure to ibuprofen in the first seven days after surgery. Patients receiving ibuprofen during the first postoperative week, but after the development of AKI, were considered non-exposed. Ibuprofen was only administered enterally at our institution.

The primary outcome was AKI within seven days after surgery. Postoperative AKI was defined according to the serum creatinine criteria of the KDIGO definition [8]. The secondary outcome was severe AKI, defined as KDIGO stage 2 and 3 AKI. AKI was not defined using the urine output criteria because frequently measured urine output values were not available. This study was limited to children older than 1 month of age because the definition of AKI in neonates is significantly different from that in older children [9].

### Statistical analysis

Continuous variables were presented as median (interquartile range [IQR]). Continuous variables were compared between groups using the Wilcoxon rank-sum test. Categorical variables were presented as numbers (percentages) and compared using Pearson’s chi-square tests. A multivariable Cox regression model with time-varying ibuprofen exposure was used to determine the association between postoperative ibuprofen use and AKI. Other relevant covariates involved in this Cox model included age at operation, sex, weight, cyanosis, prior cardiac surgery, baseline serum creatinine, serum albumin, hemoglobin, LVEF, RACHS-1 score, emergency surgery, CPB duration ≥ 120 min, and cross-clamp time. We also conducted subgroup and interaction analyses to assess the potential effect modification by age at operation, RACHS-1 score, CPB time, and baseline serum creatinine. Multivariable Cox proportional hazards regression was performed for subgroup analyses, and *P* for interaction <0.1 was considered significant. The secondary outcome was analyzed also using a multivariable Cox proportional hazards regression model with the same covariates in the primary analysis.

Two sensitivity analyses were performed. First, time-varying exposure to ibuprofen was defined as receiving the first dose of ibuprofen within 48 h after surgery to assess whether the timing of postoperative ibuprofen exposure would affect its association with AKI. Second, propensity score-matching (PSM) was used to mitigate potential confounding effects and treatment selection bias. The propensity scores were estimated by a multivariable logistic regression model. The covariates included age at operation, sex, weight, cyanosis, prior cardiac surgery, baseline serum creatinine, serum albumin, hemoglobin, LVEF, RACHS-1 score, emergency surgery, CPB duration ≥ 120 min, and cross-clamp time. Patients with and without ibuprofen treatment in the postoperative period were matched in a 1:1 ratio using greedy nearest-neighbor matching with a matching caliper of 0.2 standard deviations of the logit of the estimated propensity score. After propensity score-matching, the association between time-varying ibuprofen exposure and AKI was assessed using Cox proportional hazards regression adjusted for the same covariates in the primary analysis.

A 2-tailed *p* < 0.05 was considered statistically significant for all analyses. All statistical analyses were performed using a statistical programming language (R, version 4.1.2; R Development Core Team).

## Results

### Study population and baseline characteristics

**Figure 1.**
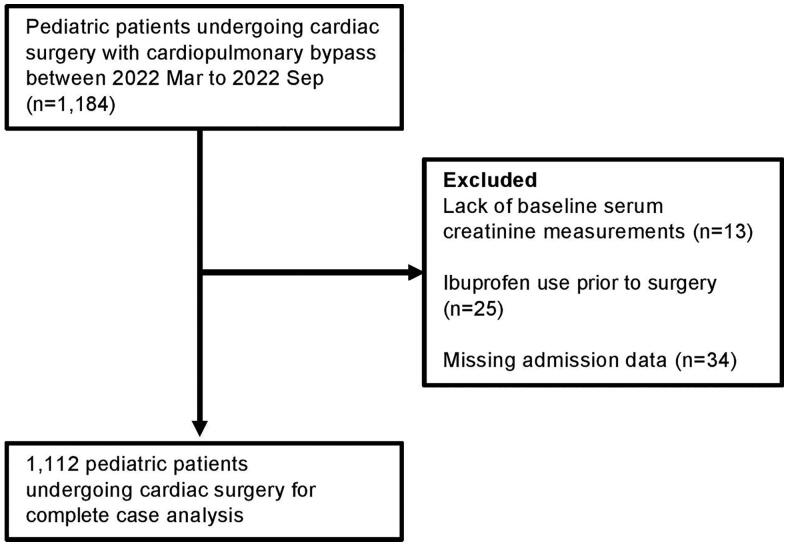
Flow diagram of the cohort study.

The final cohort included 1,112 children undergoing cardiac surgery with CPB (Figure 1). A total of 72 children were excluded due to lack of baseline serum creatinine measurements (*n* = 13), ibuprofen use prior to surgery (*n* = 25), or missing admission data (*n* = 34). In this cohort, 396 individuals (35.6%) given ibuprofen in the first 7 days after surgery were considered as ibuprofen users. Of the 716 individuals who were considered unexposed to ibuprofen after surgery, 673 were not administered ibuprofen in the first 7 days after surgery and 43 received ibuprofen after AKI. Children who did not receive ibuprofen were younger and had lower weight, fewer previous cardiac surgeries, lower RACHS-1 score, lower baseline serum creatinine, more numbers of creatinine values measured, and lower baseline hemoglobin (Table 1).

**Table 1.** Characteristics of ibuprofen exposed vs unexposed. Data are number (percentage) or median (25th–75th percentiles).

Variable	Ibuprofen *N* = 396	No Ibuprofen *N* = 716	*P* Value
Age (months), median (IQR)	38.8 (13.6–71.5)	33.6 (9.7–66.6)	0.005
Male sex, N (%)	179 (54.8)	340 (52.5)	0.465
Weight (kg), median (IQR)	14.0 (9.0–20.0)	13.0 (8.0–19.1)	0.015
Previous cardiac surgeries, N (%)	37 (9.3)	23 (3.2)	<0.001
Preoperative cyanosis, N (%)	17 (4.3)	40 (5.6)	0.349
Emergent surgery, N (%)	1 (0.3)	5 (0.7)	0.331
RACHS-1 score, N /total N (%)			0.005
1	55 (13.9)	102 (14.3)	
2	136 (34.3)	315 (44.0)	
≥3	205 (51.8)	299 (41.7)	
CPB time (min), median (IQR)	80 (53–114)	70 (47–104)	0.002
CPB time ≥120 min, N (%)	90 (22.7)	128 (17.9)	0.051
Cross-clamp time (min), median (IQR)	50 (29–76)	42 (25–67)	0.001
Baseline LVEF (%), median (IQR)	69 (65–73)	69 (65–72)	0.778
Baseline serum creatinine (mg/dL), median (IQR)	0.34 (0.27–0.43)	0.32 (0.24–0.40)	<0.001
Baseline serum albumin(g/L), median (IQR)	4.4 (4.2–4.5)	4.4 (4.1–4.5)	0.273
Baseline hemoglobin (g/dL), median (IQR)	12.4 (11.6–13.3)	12.3 (11.4–13.1)	0.011

IQR: interquartile range; RACHS: Risk Adjustment for Congenital Heart Surgery; CPB: cardiopulmonary bypass; LVEF: left ventricular ejection fraction.

### Association of ibuprofen administration with any-stage AKI

**Figure 2.**
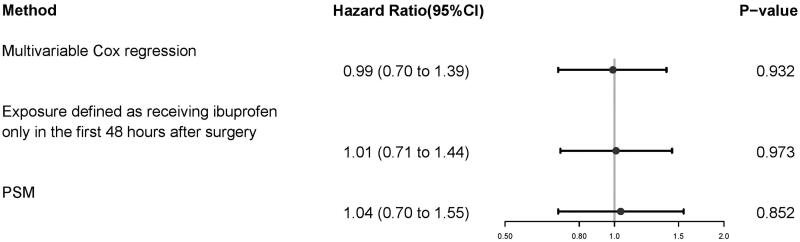
The association between ibuprofen exposure and postoperative AKI. Three different methods were used to assess the association: (1) multivariable Cox regression with time-varying ibuprofen exposure, (2) the same multivariable Cox regression model with ibuprofen exposure defined as given ibuprofen within 48 h after surgery, (3) propensity score-matching analysis. *PSM*, propensity-score matching.

In the entire cohort, 17.8% (198 of 1112) of children had any-stage AKI in the first 7 days after surgery. The incidence of any-stage AKI was lower in children who received ibuprofen (11.6% [46 of 396]) than in those who did not (21.2% [152 716]) (*p* < 0.001) (Table 1). However, there was no association between ibuprofen use and any-stage AKI after adjusting for confounders (adjusted HR, 0.99; 95% CI 0.70–1.39, *p* = 0.932) (Figure 2).

### Sensitivity and subgroup analyses

The results from the sensitivity analyses were consistent with those from the primary analysis. When we defined ibuprofen exposure as receiving the first dose within 48 h after surgery, there was also no significant association between ibuprofen use and any-stage AKI (adjusted HR 1.01, 95% CI 0.71–1.44, *p* = 0.973) (Figure 2).

After propensity score matching between the exposed and non-exposed groups, 380 pairs were obtained and the baseline characteristics were well-balanced between the two groups (Supplementary Table 1). Similarly, there was no association of use of ibuprofen with any-stage AKI (adjusted HR 1.04, 95% CI 0.70–1.55; *p* = 0.852) (Figure 2).

**Figure 3.**
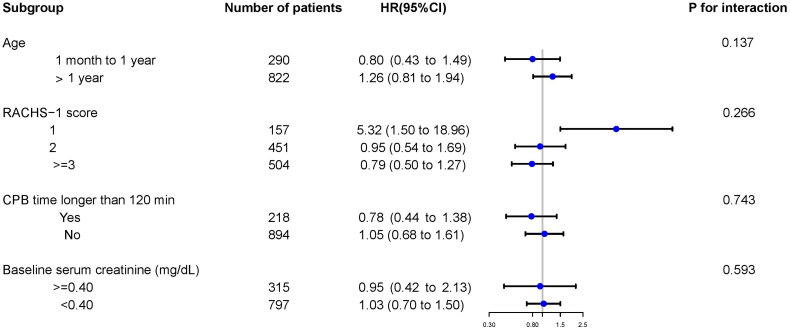
Subgroup and interaction analyses for age, RACHS-1 score, CPB time, and baseline serum creatinine. Multivariable Cox regression model with time-varying ibuprofen exposure was used to assess the association of ibuprofen exposure with AKI. *RACHS*, Risk Adjustment for Congenital Heart Surgery; *CPB*, cardiopulmonary bypass; *HR*, hazard ratio.

In each group stratified by age, RACHS-1 score, CPB time, and baseline serum creatinine, the overall results from the subgroup analyses were consistent with the main results (Figure 3). In addition, there were no significant interactions between ibuprofen exposure and any of the subgroups (Figure 3).

### Secondary outcome

Children with ibuprofen exposure were less likely to develop severe AKI than those without (0.5% [2 396] vs 5.0% [36 716], *p* < 0.001) (Table 2). After adjusting for confounders, there was a trend toward a lower risk of severe AKI in ibuprofen users (adjusted HR 0.62; 95% CI 0.24–1.62). However, this association did not reach statistical significance (*p* = 0.328).

**Table 2.** Incidence of AKI according to ibuprofen exposure.

Outcome	Ibuprofen	No Ibuprofen	*P* Value
	*N* = 396	*N* = 716	
Overall AKI	46 (11.6%)	152 (21.2%)	<0.001
Severe AKI	2 (0.5%)	36 (5.0%)	<0.001

AKI: acute kidney injury.

## Discussion

This single-center retrospective cohort study investigated the impact of ibuprofen administration on renal outcomes in pediatric patients undergoing cardiac surgery. We found that children exposed to ibuprofen after surgery were less likely to develop AKI. However, after adjusting for confounders, this association was not observed in the multivariable Cox proportional hazards regression analysis. Our findings do not support the hypothesis that exposure to ibuprofen is associated with an increased incidence of AKI after cardiac surgery in children.

In our cohort, 17.8% of pediatric patients had postoperative AKI in the first 7 days after surgery. The incidence of overall AKI reported in our study was relatively low compared to that reported in previous studies [10]. This discrepancy may be attributed to several reasons. First, we defined AKI only according to the serum creatinine criteria, as we did not have reliable urine output data for all pediatric patients to calculate their urine output rate in the postoperative period. Second, younger age is associated with an increased risk of AKI [1], the median age of the included pediatric patients in this study was relatively older than that in previous studies with high AKI incidence [10].

Although NSAIDs are defined as nephrotoxic medications according to the Nephrotoxic Injury Negated by Just-in-time Action Collaborative [11], there are controversies regarding the effect of NSAIDs on AKI in children [4–7,12–15]. In a retrospective cohort study of 154 pediatric patients undergoing cardiac surgery, the authors found that high nephrotoxin exposure was not associated with a higher risk of AKI [4]. Among the nephrotoxins assessed in this study, NSAIDs including ketorolac, aspirin, and ibuprofen were the most commonly used. However, the effect of nephrotoxic medications on AKI might be obscured by the relatively small sample size and unadjusted confounders that were risk factors of AKI. In a prospective case-control study of dehydrated children with acute gastroenteritis, ibuprofen exposure was associated with an increased risk of AKI [7]. This study also suggested that ibuprofen-associated AKI is typically mild and transient as most patients were at the ‘injury’ stage of AKI severity according to the RIFLE criteria and all AKI cases were fully recovered within 8 days [7]. In a large multicenter retrospective study of 50,420 hospitalized children from 25 participating centers, ibuprofen use was associated with an increased risk of hospital-acquired AKI in a dose-dependent manner [6]. This association was even more prominent in children of older age, those with chronic kidney disease, and those needing intensive care. Another prospective multicenter study investigated 25 children with NSAID-associated AKI and 60% of them were given ibuprofen [13]. The authors found that NSAID-associated AKI usually developed in dehydrated children, even at the recommended dosages. Of note, volume depletion is commonly observed in children undergoing pediatric cardiac surgery [16].

The results of this retrospective cohort study showed no association between ibuprofen administration and AKI after pediatric cardiac surgery in multivariable Cox regression analysis. This association was confirmed by two sensitivity analyses using a different association inference model as well as a modified exposure definition, respectively. However, we found that children exposed to ibuprofen were less likely to develop AKI in the univariate analysis. This might be due to the unbalanced distribution of baseline characteristics between the exposed and unexposed groups. We did not detect a nephrotoxic effect of ibuprofen in the study population, which has been reported in previous studies [6,7]. This difference might be due to the fact that the participants in our study were relatively young. Subgroup analysis in a previous large cohort study suggested that ibuprofen exposure was associated with a significantly higher risk of AKI in children older than 10 years; however, this association was not observed in those younger than 1 year [6]. In general, the differences in the effect of ibuprofen on renal function among different studies might be due to the differences in patient characteristics and timing and dosages of ibuprofen administration.

There is no evidence suggesting that ibuprofen provides renoprotection. However, recent studies showed that ibuprofen has a protective effect on neurologic and myocardial injuries [17–21]. The anti-inflammatory property of ibuprofen found in these studies might also affect renal function in cardiac surgical patients since postoperative AKI is partially mediated by inflammation caused by surgical insult and the use of CPB [22].

Although this investigation is a relatively large study for the evaluation of the potential nephrotoxicity of ibuprofen in the pediatric population undergoing cardiac surgery, some limitations exist in the study. First, as a retrospective observational study, the baseline characteristics differed between the exposed and unexposed groups. Second, we did not perform a dose-response analysis as the accurate daily doses of ibuprofen were unavailable in our database. Third, we did not use both the urine output and creatinine criteria for the diagnosis of postoperative AKI. Fourth, residual bias might exist due to unmeasured confounders, such as the use of other nephrotoxic medications and the presence of volume depletion. Fifth, information regarding the indications for ibuprofen administration was not available owing to the retrospective design, which might have caused indication bias.

## Conclusions

Postoperative ibuprofen use in pediatric patients undergoing cardiac surgery was not associated with an increased risk of AKI after adjusting for confounders. More studies are needed as ibuprofen remains a potentially nephrotoxic medication.

## Ethics approval

The Fuwai Hospital Institutional Review Board in China approved this study.

## Supplementary Material

Supplemental Material

## Data Availability

The data of this study are available from the corresponding author, Jianhui Wang, upon reasonable request.
