# Supplementary material for: Association between postoperative ibuprofen exposure and acute kidney injury after pediatric cardiac surgery
Source: Ren Fail. 2024 Feb 19;46(1):2318417. doi: 10.1080/0886022X.2024.2318417 (PMC10880564; doi:10.1080/0886022X.2024.2318417)
Supplement: Supplemental Material [file IRNF_A_2318417_SM2477.pdf]

Variable	Pre-PSM ASD	Post-PSM ASD
Age	0.163	0.023
Male sex	0.046	0.042
Weight	0.169	0.041
Previous cardiac surgeries	0.255	0.035
Preoperative cyanosis	0.060	0.038
Emergent surgery	0.065	0.073
RACHS-1 score	0.172	0.036
CPB time ≥120 min	0.121	0.020
Cross-clamp time	0.177	0.028
Baseline LVEF	0.060	0.027
Baseline serum creatinine	0.204	0.011
Baseline serum albumin	0.087	0.053
Baseline hemoglobin	0.153	0.002

Supplementary Table 1. Comparison of the extent of covariate imbalance by absolute standardized differences before and after propensity-score matching. *ASD,* absolute standardized difference; *RACHS*, Risk Adjustment for Congenital Heart Surgery; *CPB*, cardiopulmonary bypass; *LVEF*, left ventricular ejection fraction
